# A Cell Line for Detection of Botulinum Neurotoxin Type B

**DOI:** 10.3389/fphar.2017.00796

**Published:** 2017-11-09

**Authors:** Aleksander Rust, Ciara Doran, Rosalyn Hart, Thomas Binz, Paul Stickings, Dorothea Sesardic, Andrew A. Peden, Bazbek Davletov

**Affiliations:** ^1^Department of Biomedical Sciences, University of Sheffield, Sheffield, United Kingdom; ^2^Institut für Zellbiochemie, Medizinische Hochschule Hannover, Hannover, Germany; ^3^Division of Bacteriology, National Institute for Biological Standards and Control, Medicines and Healthcare Product Regulatory Agency, Potters Bar, United Kingdom

**Keywords:** botulinum neurotoxin type B, botulinum neurotoxin-sensitive cell line, SiMa neuroblastoma, VAMP, tetanus, luciferase, Myobloc, 3Rs

## Abstract

Botulinum neurotoxins (BoNTs) type A and type B are commonly used as biopharmaceutics for neurological diseases, uniquely allowing months-long paralysis of target muscles. Their exquisite neuronal specificity is conferred by a multistep process of binding, internalization, cytosolic escape and cleavage of the neuron-specific proteins, SNAP-25 and vesicle-associated membrane proteins (VAMPs), ultimately to inhibit secretion of neurotransmitters. Currently the mouse lethality bioassay is the only available method for quality control testing of VAMP-cleaving botulinum products. Refined assays for botulinum product testing are urgently needed. Specifically, *in vitro* replacement assays which can account for all steps of BoNT intoxication are in high demand. Here, we describe a novel SiMa cell-based approach where re-engineering of the VAMP molecule allows detection of all BoNT/B intoxication steps using a luminescent enzymatic reaction with sensitivity comparable to mouse LD_50_ bioassay. The presented one-step enzyme-linked immunosorbent assay meets 3Rs (replacement, reduction, and refinement of the use of animals) objectives, is user-friendly and will accelerate development of new botulinum drugs. The sensitive enzymatic reporter cell line could also be adapted for the detection of toxin activity during the manufacture of botulinum and tetanus vaccines.

## Introduction

Botulinum neurotoxins (BoNTs) are produced by anaerobic bacteria of the genus *Clostridium* and are responsible for the deadly disease called botulism manifested by neuromuscular paralysis (Erbguth and Naumann, 1999; Schiavo et al., 2000; Montecucco and Molgó, 2005). In the last three decades, BoNTs have been utilized widely in many medical applications when injected locally and in small doses (Davletov et al., 2005; Chaddock and Marks, 2006; Foster et al., 2006). A typical BoNT is expressed by bacteria as a single chain precursor protein that is processed into two polypeptide chains – a 100 kD heavy chain consisting of the receptor-binding domain and the translocation domain which is linked via a disulphide bond to 50 kD light chain, a SNARE protease (Lacy et al., 1998; Chaddock and Marks, 2006; Binz and Rummel, 2009). Among seven commonly known BoNT serotypes (A–G) BoNT/A, C, and E proteolyse SNAP-25, while BoNT/B, D, F, and G cleave vesicle-associated membrane proteins (VAMPs) also known as synaptobrevins (Lacy and Stevens, 1999; Schiavo et al., 2000; Rummel et al., 2004; Antonucci et al., 2008; Rossetto and Montecucco, 2008; Binz et al., 2010). In order to reach their intraneuronal substrates, BoNTs first bind neuronal surface gangliosides and then a synaptic vesicle protein (synaptotagmin or SV2) on the presynaptic membrane for subsequent internalization (Montecucco and Schiavo, 1994; Binz and Rummel, 2009). Once the internalized vesicle acidifies, the botulinum translocation domain changes conformation to form a putative protein transduction channel that enables translocation of the protease into the cytosol following reduction of the disulphide bond (Koriazova and Montal, 2003; Puhar et al., 2004; Pirazzini et al., 2013).

Pharmaceutical BoNT/A (e.g., Botox^®^) and BoNT/B (e.g., Myobloc^®^, Neurobloc^®^) products are mainly licensed for the treatment of neuromuscular spasms, but their use is expanding to other conditions such as hyperhidrosis, bladder dysfunction, spasmodic dysphonia, sialorrhoea, anal fissures, piriformis syndrome, various pain conditions, and cosmetic applications. As a “biologic” medicine, each new batch of BoNT is considered a new product and must undergo rigorous potency, quality and safety testing before market release. In addition assays are needed for confirmation of natural cases of botulism, in food testing and prevention of both human- and animal-targeted bioterrorism. Currently the “gold standard” toxicity test is the mouse LD_50_ lethality bioassay. This has many serious disadvantages including imprecision, necessitating the use of many laboratory animals (Sesardic et al., 2003), along with animal suffering due to the lethal endpoint, high operational cost, and lack of specificity since all BoNT serotypes will cause similar muscular paralysis. Therefore, more precise replacement assays that follow the principles of the 3Rs (reduction, replacement, and refinement of animal use in research) are urgently needed. An ideal replacement assay must faithfully represent all the biological steps of BoNT action. Such a replacement method has been recently developed for BoNT/A type products where SiMa neuroblastoma cell line is being adapted as cell based potency assay for Botox^®^ (Fernández-Salas et al., 2012). The assay format is a sensitive sandwich enzyme-linked immunosorbent assay, ELISA, detecting a stable BoNT/A-cleaved SNAP-25 product. To date, however, there are no cell-based assays for testing BoNT/B which proteolyses VAMP molecules. Here, we describe an *in vitro* assay using the SiMa cell line (Marini et al., 1999) that has been engineered to carry a stabilized VAMP molecule to report the BoNT/B activity via a luminescent reaction.

## Results

### Introduction of Stabilized VAMP2 into the SiMa Neuroblastoma Cell Line for BoNT/B Detection

**FIGURE 1:**
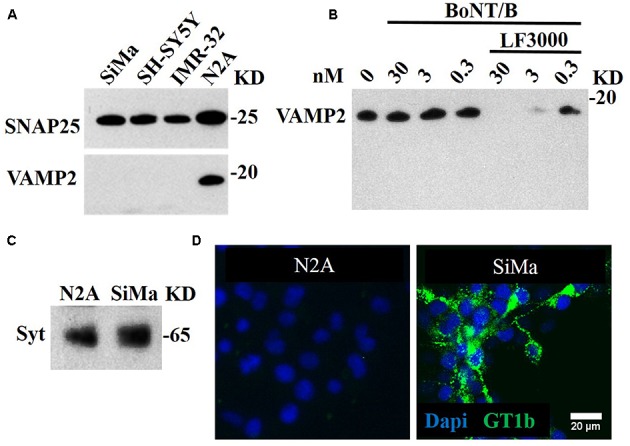
BoNT/A-sensitive SiMa neuroblastoma cell line expresses neuronal gangliosides and synaptotagmin but not BoNT/B substrate VAMP2. **(A)** Three neuroblastoma cells (SiMa, SH-SY5Y and IMR-32) express the BoNT/A substrate SNAP25 but not the BoNT/B target VAMP2. Only the N2A neuroblastoma cell line expresses both SNAP25 and VAMP2. **(B)** BoNT/B does not cleave VAMP2 in N2A cells at indicated concentrations. Transduction of BoNT/B into N2A cells using Lipofectamine LF3000 (LF3000) allows VAMP2 cleavage. Note the absence of VAMP fragments at all BoNT/B concentrations. **(C)** A polyclonal antibody against synaptotagmin 1 reveals its presence in the SiMa and N2A cell lines. **(D)** The SiMa but not N2A cell line expresses neuronal ganglioside GT1b as revealed by specific anti-GT1b antibody.

We tested several candidate neuronal cell lines – SiMa, SH-SY5Y, IMR-32, and N2A – for expression of the BoNT/B target, VAMP2. **Figure 1A** illustrates that only the N2A cell line expressed VAMP2. We tested N2A cells for sensitivity to BoNT/B using Western immunoblotting for cleavage of VAMP2. **Figure 1B** shows that no cleavage was detected when the cells were incubated with concentrations as high as 30 nM BoNT/B. However, when BoNT/B entry was facilitated by Lipofectamine 3000 (Rust et al., 2016), we observed dose-dependent disappearance of VAMP2, indicating that BoNT/B can cleave VAMP2 but is unable to enter N2A cells. Following BoNT/B-induced cleavage there were no VAMP2 breakdown products (**Figure 1C**) most likely due to their immediate degradation by intracellular clearance mechanisms (Foran et al., 2003b). Degradation of the fragments of VAMP2 precludes the direct detection of botulinum-cleaved fragment necessitating novel synthetic approaches.

Recently, Fernández-Salas et al. (2012) developed a cell-based assay specifically for testing the stability and potency of BoNT/A using SiMa neuroblastoma cells. We have also recently reported that SiMa cells can be used to detect BoNT type C activity in the picomolar range (Rust et al., 2016). We therefore tested this neuroblastoma cell line for the presence of BoNT/B receptors – synaptotagmin 1 and the ganglioside GT1b. Immunoblotting revealed that both N2A and SiMa cells express synaptotagmin 1. In contrast, **Figure 1C** shows that SiMa but not N2A cells are immunopositive for the complex ganglioside GT1b. This is in agreement with previous studies showing that N2A cells lack the neuronal gangliosides necessary for BoNT entry (Yowler et al., 2002) and protein transduction methods are required to introduce the botulinum proteolytic domains (Arsenault et al., 2014).

**FIGURE 2:**
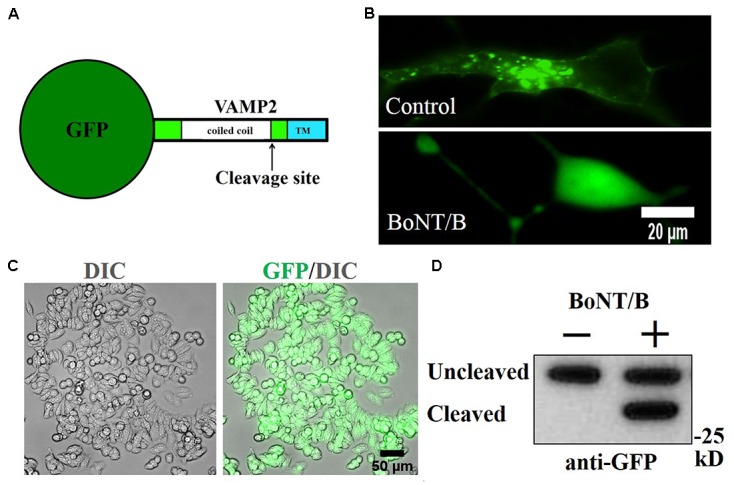
Generation of GFP-VAMP2 SiMa cell line using viral transduction. **(A)** Schematic showing the fusion protein containing GFP inserted at the amino terminus of VAMP2. TMR, transmembrane region. **(B)** GFP-VAMP2 localizes to vesicular structures (top) and is released into the cytosol (bottom) upon incubation with BoNT/B (30 nM). **(C)** Robust expression following viral transduction and puromycin selection is evident in GFP and differential interference contrast (DIC) merged images. **(D)** Stable GFP-VAMP2 cleavage product can be detected following incubation with BoNT/B (30 nM). Immunoblotting was performed using a GFP antibody.

Since both N2A and SiMa express some of the necessary components for BoNT/B sensitivity, but lack others, we postulated that we could engineer a BoNT/B sensitive cell line by a synthetic gene approach. Ganglioside biosynthesis requires a complex, multigenic mechanism (Yu et al., 2011), making N2A less amenable to engineering. We hypothesized that SiMa cells could report BoNT/B activity if we introduce an engineered VAMP2 exogenously. Therefore, we generated a SiMa cell line stably expressing GFP-VAMP2 using retroviral transduction followed by puromycin selection. We used a fusion protein construct that has GFP inserted at the amino terminus of VAMP2 (**Figure 2A**) such that GFP will be anchored to vesicles facing the cytosol. At high magnification, GFP-VAMP2 exhibits a punctate distribution in SiMa cells, consistent with the predicted localisation to vesicular structures (**Figure 2B**, upper). When the cells were incubated with BoNT/B (30 nM), the pattern of GFP expression became diffuse (**Figure 2B**, bottom) consistent with cleavage of VAMP2 by the botulinum protease followed by the release of GFP from vesicles into the cytosol. Following puromycin selection all of SiMa cells were positive for GFP-VAMP2, as evidenced by fluorescent microscopy (**Figure 2C**). The native VAMP2 is quickly degraded in neurons once cleaved at a single position by BoNTs. Importantly, immunoblotting revealed that a band corresponding to the cleaved GFP-VAMP2 product is completely stable in the neuroblastoma cell line (**Figure 2D**), indicating that the GFP added to the N-terminal end of VAMP2 somehow prevents degradation offering the possibility to measure the botulinum-cleaved VAMP2 product.

### Generation of a BoNT/B-Sensitive Enzymatic Reporter Cell Line

**FIGURE 3:**
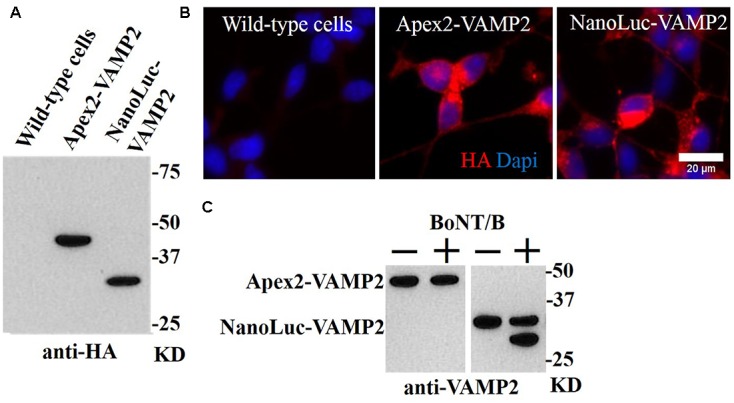
Generation of stable SiMa cell lines with VAMP2 fusions to peroxidase APEX2 and luciferase NanoLuc. **(A)** Immunoblot showing expression of APEX2- and NanoLuc-VAMP2 constructs in SiMa cells with expected molecular weights of ∼45 kD and ∼30 kD. Both constructs carry the HA tag as evidenced by immunoblotting. **(B)** The fusion proteins exhibit robust expression as revealed by immunocytochemical analysis using antibodies against the HA-tag. **(C)** Immunoblot demonstrating that the APEX2-VAMP2 reporter molecule is resistant to cleavage by BoNT/B (10 nM), whereas VAMP cleavage is detectable in the differentiated NanoLuc-VAMP2 cells. The arrow indicates the cleaved product.

While our GFP-VAMP2 cell line offers a possibility to develop a “high content” analysis assay using automated microscopy or sandwich ELISA-based detection as described for BoNT/A-cleaved SNAP25 (Fernández-Salas et al., 2012), we hypothesized that a direct linkage of an enzyme to the VAMP molecule will allow faster and easier detection of BoNT/B activity if we can capture the proteolytic product on microplates. One-step capture immunoassay utilizing enzymatic reporter molecules offers the dual advantage of fewer washing and incubation steps coupled with superior sensitivity due to signal amplification (Boute et al., 2016). Using viral transduction we generated two stable cell lines expressing either peroxidase- or luciferase-linked VAMP2 products, namely APEX2-VAMP2 and NanoLuc-VAMP2, respectively. Both constructs also carry a hemagglutinin-related HA tag allowing a convenient immunological detection (**Figure 3A**). Immunocytochemistry revealed that both constructs exhibited robust expression inside SiMa cells (**Figure 3B**). The engineered cells were then differentiated and incubated with BoNT/B (10 nM) for 48 h before evaluation of the VAMP2 cleavage by immunoblotting with an antibody against VAMP2. Two bands, corresponding to cleaved and uncleaved VAMP2 molecules, were readily detectable in the NanoLuc-VAMP2 cell line (**Figure 3C**). Curiously, we could not detect any cleavage in the APEX2-VAMP2 cell line (**Figure 3C**), which may be explained by steric hindrance introduced by a relatively large peroxidase enzyme.

### BoNT/B Detection Using a Cleavage-Specific Antibody and the NanoLuc-VAMP Assay

**FIGURE 4:**
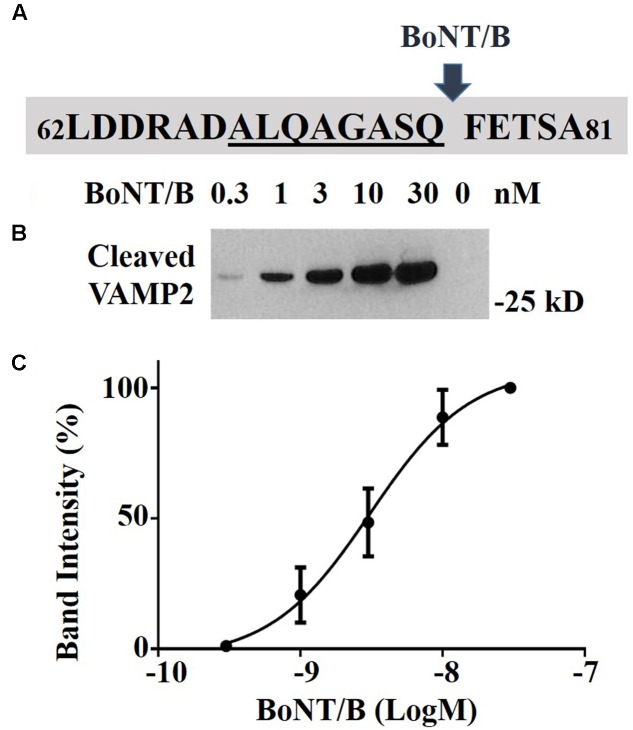
Detection of BoNT/B activity in NanoLuc-VAMP2 SiMa cells using specific BoNT/B-cleaved VAMP2 antibody. **(A)** The VAMP2 amino acid sequence used for generation of the rabbit polyclonal antibody is underlined. The arrow indicates the cleavage site by BoNT/B. **(B)** Immunoblot showing dose-dependent increase of BoNT/B-cleaved NanoLuc-VAMP2 product using the cleaved VAMP2 antibody. Engineered SiMa cells were incubated for 60 h in the presence of indicated concentrations of BoNT/B followed by SDS–PAGE and immunoblotting. **(C)** Graph showing quantification of dose-dependent increase in cleaved VAMP2 immunoblotting signal in response to BoNT/B with sensitivity in the low picomolar range (*n* = 3 ± SD).

The stabilized BoNT/B-cleaved substrates offer the opportunity to detect the BoNT/B-cleaved fragment using cleavage-specific antibody directed against the cleaved end of the VAMP2 molecule. We generated a rabbit BoNT/B-cleaved VAMP antibody using the peptide antigen sequence shown in **Figure 4A**. We tested the ability of the cleaved VAMP antibody to detect the cleaved product following application of BoNT/B to the engineered SiMa cells. **Figure 4B** shows that application of increasing doses of BoNT/B led to increased immunoblotting signal corresponding to the 28 kD NanoLuc-VAMP2 cleavage product being detected by the cleaved VAMP antibody. No signal was detectable in the vehicle control sample, consistent with antibody specificity for the cleaved VAMP product only. **Figure 4C** shows densitometry quantification of dose-dependent sensitivity of immunoblotting using the cleaved VAMP2 antibody with limit of detection being 300 pM BoNT/B in differentiated SiMa cells.

**FIGURE 5:**
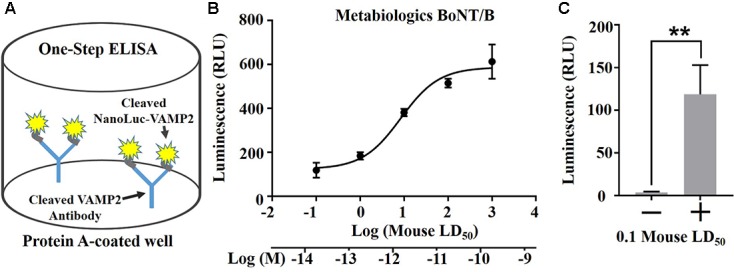
One-step ELISA assay using the NanoLuc-VAMP2 construct. **(A)** Schematic (left) showing the principle of one-step ELISA. The cleaved VAMP2 antibody was immobilized on Protein A plates for capture of the BoNT/B-cleaved VAMP2 construct. **(B)** Graph showing the dose-dependent detection of NanoLuc-VAMP2 cleavage by Metabiologics BoNT/B (*n* = 3 ± SD). **(C)** The NanoLuc assay allows detection of the VAMP cleavage by BoNT/B at 0.1 mouse LD_50_ above the baseline (*n* = 3 ± SD, ^∗∗^*p* < 0.01, Student’s *t*-test).

Next we explored luminescent detection which is afforded by the NanoLuc-VAMP2 construct using a highly sensitivity NanoGlo reaction and commercial BoNT/B with a known mouse LD_50_ activity (Metabiologics, United States). We immobilized the cleaved-VAMP2 antibody on Protein A-coated 96-well plates and evaluated the detection range of BoNT/B activity in one-step ELISA format (**Figure 5A**). BoNT/B with known LD_50_ activity was applied to the engineered SiMa cells at a wide range of concentrations for 60 h and the generated NanoLuc-VAMP2 fragment was captured on the cleaved-VAMP2 antibody plates. Following washing the wells were filled with NanoGlo solution and luminescent signal was read in a plate luminometer. The one-step ELISA showed NanoLuc-VAMP detection sensitivity similar to the mouse LD_50_ specified by the manufacturer (**Figure 5B**). Importantly, BoNT/B activity was detectable in one-step ELISA assay even at the lowest dose tested of 0.1 mouse LD_50_, which corresponds to ∼20 fM BoNT/B (**Figure 5C**).

## Discussion

Despite their distinction as the most lethal toxins known to man, BoNTs are increasingly being harnessed to treat a range of previously intractable neuromuscular and neurological conditions. The neuronal specificity of the seven different BoNT serotypes is an active area of investigation, however, they all share a general mechanism of action, which has evolved to specifically target the nervous system. This comprises toxin binding to a cell surface receptor, internalization of the toxin-receptor complex, light chain translocation into the cytoplasm and proteolytic cleavage of a SNARE protein, ultimately to inhibit the exocytosis machinery (Foran et al., 2003a). A wide variety of biochemical *in vitro* assays for BoNT toxicity testing have been developed to reduce and replace current *in vivo* lethality assays. Single parameter assays, e.g., SNARE cleavage assay or ganglioside binding, are unfortunately unable to distinguish between biologically active and inactive BoNTs. Recently, a dual parameter BINACLE (binding and cleavage) assay has been reported to offer detection limit of less than 1 pg/ml, equivalent to less than 0.1 mouse LD_50_ units/ml of BoNT/B (Wild et al., 2016). One specific limitation is that the biochemical BINACLE assay takes into account only receptor binding and the VAMP cleavage steps of intoxication without probing the cellular translocation function and therefore cannot fully replace the mouse bioassay in the quality control of pharmaceutical products. It is generally agreed that in the context of pharmacological botulinum product testing, a cell-based assay encompassing all steps of BoNT action shall be developed to replace the rodent lethality assays. The ‘gold standard’ mouse bioassays are exquisitely sensitive to BoNT/A and BoNT/B (10–30 pg/ml) (Wictome et al., 1999; Ferreira et al., 2004) and it has been a great challenge in the field to find alternatives. Recently, cell-based assays have been approved by regulatory authorities for pharmaceutical BoNT/A testing (Fernández-Salas et al., 2012; Merz, 2015). However, currently there are no equivalent cell assays for BoNT/B. For BoNT/B, the best reported EC_50_ values in primary cells were reported in rat spinal cord neurons with an EC_50_ of ∼29 mouse LD_50_ units (Whitemarsh et al., 2012), which is only in a low pM range. One major drawback of using primary cells is that they must be matured in culture for 2 weeks. Moreover, they are not favored by regulatory agencies because of their inherent biological variability (Adler et al., 2010). Human induced pluripotent stem cells (hiPSCs) derived neurones can exceed the sensitivity of primary cells, however, the best reported sensitivity for BoNT/B in hiPSCs was ∼16 mouse LD_50_ units (Whitemarsh et al., 2012). Moreover, if purchased undifferentiated, hiPSCs require 3–4 weeks for maturation with considerable hands-on manipulation and specialized expertise. It is possible to purchase pre-differentiated hiPSCs, but, currently, costs are prohibitive for wide-spread adaption. Western immunoblotting detecting disappearance of native neuronal VAMP2 is also not optimal for accurate quantification. Indeed, detection of appearance of cleavage product is much better suited for assay development as demonstrated by SNAP25 cleavage assay in the case of BoNT/A (Fernández-Salas et al., 2012). Here, we described a novel approach for BoNT/B detection assay using a continuous cell line carrying a stabilized VAMP2 cleavage reporter. Our NanoLuc-VAMP2 SiMa neuroblastoma assay far exceeds the rat spinal cord cell sensitivity using a very simple 3-day differentiation protocol. It appears that the differentiated SiMa neuroblastoma cells are sensitive to BoNTs (Fernández-Salas et al., 2012; Rust et al., 2016; Bak et al., 2017) due to the high level of expression of neuron-specific GT1b ganglioside (**Figure 1D**) which is essential for the initial binding of the toxins (Montecucco and Schiavo, 1994; Binz and Rummel, 2009). If needed, sensitivity could be improved further by stably transfecting into the NanoLuc-VAMP2 SiMa neuroblastoma cells the mouse synaptotagmin 2 which has 10 times higher affinity to BoNT/B compared to human synaptotagmin (Strotmeier et al., 2012). Subcloning of the parental SiMa cell line and clonal screening for highest sensitivity to BoNT/B offers a possibility to further enhance the one-step ELISA for BoNT/B detection. Finally, optimisation of SiMa differentiation protocol using well established methods could be also tested to increase BoNT/B sensitivity if required.

In summary, the NanoLuc-VAMP reporter cell assay has the potential to offer an accurate replacement for the BoNT/B mouse bioassay in a homogeneous cell line with excellent sensitivity. The main advantage of the described method is that unlike in neurons, the NanoLuc-VAMP2 cleavage product evades degradation following cleavage by BoNT/B offering a highly specific and sensitive enzymatic read-out of BoNT/B activity. The assay may well be suited for measuring potency of pharmaceutical BoNT/B products and antitoxin antibodies in toxin neutralization testing. The presented assay could be adapted to other VAMP2-cleaving BoNT serotypes and also tetanus toxin (Schiavo et al., 2000) to replace the animal lethality tests used during production of botulinum and globally essential tetanus vaccines.

## Materials and Methods

### Cell Culture, Differentiation, and Generation of VAMP2 SiMa Cell Lines

SiMa cells (DSMZ) were grown in RPMI media (Life Technologies) supplemented with 10% fetal bovine serum (FBS) (Life Technologies). IMR32 and N2A cells (Sigma) were grown in DMEM media (Life Technologies) with 10% FBS. SH-SY5Y cells (Sigma) were grown in 1:1 mix of MEM (Life Technologies) and F12 nutrient mix (Life Technologies) supplemented with 15% FBS and 1% non-essential amino acids (NEAA) (Life Technologies). Cells were maintained at 37°C, 5% CO_2_. Cell lines were kept frozen after receiving from supplier and were not used for more than 20 passages (10 weeks).

For differentiation, plates were pre-coated with 10 μg/ml laminin (Sigma) and left at 37°C for at least 1 h before washing twice with phosphate buffer saline (PBS). Wild-type and modified SiMa cell lines were seeded at a density of 1 × 10^4^ cells per well in 96-well plates or 1 × 10^5^ cells per well in 48-well plates and differentiated for 72 h. SH-SY5Y, IMR-32, and N2A cells were seeded at 5 × 10^3^ cells for 96-well plates and 2 × 10^4^ cells for 48-well plates and differentiated for 144 h. For experiments with all wild-type neuroblastoma cell lines and modified SiMa cells, the following differentiation medium was used: RPMI, 1X B-27 (Life Technologies), 1 mM HEPES, pH 7.2 (Fisher), 1% NEAA and 10 μM all-*trans*-retinoic acid (Sigma). SiMa and modified SiMa were differentiated for 72 h.

GFP-VAMP2, APEX2-VAMP2, and NanoLuc-VAMP2 were generated and cloned into the retroviral expression vector pQCXIP (Clontech). The constructs were transduced into SiMa cells using VSV-G pseudotyped viral particles produced in HEK293T cells (Swift et al., 2001). Stable expression of the reporter constructs was achieved by selecting the transduced cells with 1 μg/ml puromycin.

### Toxins

Recombinant BoNT/B was expressed in *Escherichia coli* using pBoNTBs-throm (BoNT/B gene cloned from strain Okra, subtype B1). The plasmid is a derivative of pBoNTBs (Rummel et al., 2004) encoding a thrombin cleavage site (TKSLVPRGS) integrated between the light and heavy chains at position K441–A442. BoNT/B was purified by means of its C-terminal Strep-tag and activated using thrombin. Coomassie stained protein had a purity of >95% and a degree of activation of >98%. Recombinant BoNT/B was delivered into N2A cells using Lipofectamine 3000 (Life Technologies) as described previously (Arsenault et al., 2014). For direct comparison of our method with the mouse lethality assay we used BoNT/B (Metabiologics, United States) with a molecular weight of 150 kD and a known specific activity of 1.25 × 10^8^ mouse LD_50_/mg as defined by the manufacturer. Handling procedures for BoNT/B (including the use of double gloves, avoidance of needles, class 2 safety cabinet) were approved by the Health and Safety Committee of the University of Sheffield. On completion of experiments, BoNT/B and BoNT/B-contaminated samples were inactivated by an acid/detergent mixture followed by incineration.

### Antibody Generation and Western Immunoblotting

The rabbit polyclonal antibody to BoNT/B-cleaved VAMP2 was made and affinity purified using the peptide antigen sequence K- ALQAGASQ (Davids Biotechnologie, Germany). Immunoblotting was performed as described previously (Arsenault et al., 2014). Briefly, cells were lysed in sample buffer (56 mM sodium dodecyl sulfate, 62.5 mM Tris-HCl pH 6.8, 1.6 mM EDTA, 6.24% glycerol, trace bromophenol blue, 1 mM MgCl_2_ and 0.1% benzonase). Protein concentration was measured using the DC assay (Bio-Rad) according to manufacturer’s instructions. Lysates were run on 12% Bis-Tris sodium dodecyl sulfate-polyacrylamide gel electrophoresis (SDS–PAGE) gels (Invitrogen) and protein was transferred to a polyvinylidene difluoride membrane (Bio-Rad) before probing with antibodies. Primary antibodies used were SNAP25 (in-house, 1:4000), VAMP2 (Clone 69.1, Synaptic Systems; Clone ab181869, Abcam), synaptotagmin 1 (in-house, 1:2000), GFP [in-house, 1:2000 (Gordon et al., 2017)]. Following incubation with peroxidase conjugated sheep anti-mouse or donkey anti-rabbit secondary antibodies (both 1:24000, GE Healthcare), proteins were visualized using the SuperSignal West Dura ECL reagent (Thermo Scientific) by X-ray film exposure with signals quantified using ImageJ after film scanning.

### Immunocytochemistry and Live Cell Imaging

For immunocytochemistry and live cell imaging, cells were seeded in 96-well μClear plates. For live imaging, 1 μg/ml Hoechst 33342 stain (Life Technologies) was added for nuclear staining. For immunocytochemistry, the cells were washed with PBS, fixed in 4% paraformaldehyde and then permeabilised using PBS containing 0.1% Triton X-100. Plates were then incubated in a blocking solution (8 g BSA, 8 ml fish gelatine, 0.4 ml Tween-20, 40 ml 10x PBS, and 8 ml normal goat serum in 400 ml) for 1 h before addition of an rabbit anti-HA antibody (R&D Systems, 1:2000) or mouse anti-GT1b antibody (Millipore, 1:500) and incubation for 2 h at 21°C. Following washing in PBS, cells were incubated with DAPI stain (Sigma, 1:5000) and the Alexa Fluor 594 conjugated anti-rabbit or anti-mouse secondary antibodies (Life Technologies) for 45 min. Cells were washed three times in PBS before imaging with a digital fluorescence microscope (Leica Microsystems) and a 40× objective.

### Luciferase Assay

Cell extractions were performed on ice. Cells were incubated in PBS containing 0.5% Triton-X100 and 1x SigmaFast protease inhibitor (40 μl per well) for 10 min, then detached from the well using a cell scraper. The suspension was incubated in a microtube for 20 min with vortexing every 4 min. The suspension was then centrifuged at 14,000 rpm at 4°C for 15 min and the supernatant was removed for storage at -20°C until the ELISA.

For the one-step ELISA, microplate washes were performed on a rocker (three times for 5 min at each washing stage) using PBS and 0.05% Tween wash buffer (100 μl). Protein A coated 96 well plates (Thermo Fisher Scientific, UK) were incubated with affinity purified anti-cleaved VAMP2 antibody (1 μl in 50 μl PBS) and incubated overnight at 4°C. The plates were washed and blocking solution (100 μl) containing PBS and 1% BSA was added to the well and incubated for 60 min on a rocker at 21°C. Following washing, 45 μl cell sample was added to 120 μl wash buffer and dispensed into two wells (50 μl each). Following a 90 min incubation the microplate wells were washed and then incubated with 50 μl NanoGlo solution (Promega, 4% in PBS) for 5 min in the dark. Luminescence was read on a Fluoroskan plate reader (Labsystems). Luminescence values for untreated and BoNT/B-treated NanoLuc-SiMa cells were compared using the Student’s unpaired two-tailed *t*-test, where ^∗^*p* ≤ 0.05 and ^∗∗^*p* ≤ 0.01.

## Author Contributions

AR prepared Nano-Luc VAMP2 cells and developed one-step ELISA assay, CD prepared GFP-VAMP2 cells, evaluated SiMa cell sensitivity to BoNT/B and prepared the final figures, RH evaluated VAMP2 cleavage in N2A cells, AP designed and prepared reporter VAMP2 viruses, TB prepared recombinant BoNT/B, PS and DS supplied affinity-purified cleaved VAMP2 antibody and native toxin material. All authors contributed to the preparation of the text. BD planned the experiments, analyzed the data and wrote the final version of the manuscript.

## Conflict of Interest Statement

The authors declare that the research was conducted in the absence of any commercial or financial relationships that could be construed as a potential conflict of interest. The authors declare that a patent application relating to this work has been filed.
